# Experimental and Numerical Study of Thermal Residual Stresses on Multimaterial Adherends in Single-Lap Joints

**DOI:** 10.3390/ma15238541

**Published:** 2022-11-30

**Authors:** Beatriz D. Simões, Paulo D. P. Nunes, Farin Ramezani, Ricardo J. C. Carbas, Eduardo A. S. Marques, Lucas F. M. da Silva

**Affiliations:** 1 Institute of Science and Innovation in Mechanical and Industrial Engineering (INEGI), Rua Dr. Roberto Frias, 4200-465 Porto, Portugal; 2 Departamento de Engenharia Mecânica, Faculdade de Engenharia, Universidade do Porto, Rua Dr. Roberto Frias, 4200-465 Porto, Portugal

**Keywords:** composite structures, fibre metal laminates, adhesive bonding, finite element analysis

## Abstract

The presence of residual stresses in composite materials can significantly affect material performance, especially when integrated in bonded joints. These stresses, often generated during the cure process, can cause cracking and distortion of the material, and are caused by differences in the coefficients of thermal expansion or cure shrinkage. In the current research, multimaterial adherends combining carbon-fibre-reinforced polymer (CFRP) and aluminium in a single-lap joint (SLJ) configuration are analysed, allowing us to understand the effect of the thermal residual stresses, developed during the curing process, in the overall performance of the joints. A numerical model resorting to a finite element analysis (FEA) is developed to assess and predict the behaviour of the joints. The use of FML (fibre metal laminates) was found to significantly improve the strength of the joints, as well as the failure mode. The proposed geometry performed similarly to the comparable FML geometry, in addition to a decrease in the joint weight.

## 1. Introduction

Composite structures have become progressively more popular [1] as their unique characteristics permit the development of lightweight, high-performance structures [2]. These materials present unique characteristics such as high specific strength and high specific stiffness to weight, superior fatigue behaviour, and outstanding corrosion resistance [3]. Specifically, carbon-fibre-reinforced polymers (CFRP) exhibit high strength and stiffness while maintaining low density [4], which makes them eligible to perform functions in structural parts of different configurations [5]. When integrated into structures, the preferred methods of joining remain, to this day, welding, bolting, and riveting [6,7,8,9]. However, these procedures have several key disadvantages which make them unsuitable for connecting this type of material, as these joint designs were mostly developed for metals [7]. These options present difficulties when joining dissimilar materials [8,9,10] and, specifically in the case of fasteners and rivets, the weight of the structure is increased [7]. Additionally, due to the introduction of holes, there is a local stress concentration, which hinders joint performance, as composites are very sensitive to notches [11,12]. Given this scenario, the usage of adhesive joints has been established as a viable alternative for joining these materials [10,13,14]. These joints are suitable due to their capacity to bond structures with multiple materials [14], maintaining high joint strength, as well as a smoother stress distribution [11,14] than the alternative joining processes. Furthermore, if a correct joint design is guaranteed, they must have a good energy absorption capacity and good damping, as well as prevent the entry of corrosive agents and humidity [15].

Fibre-reinforced polymers (FRP) are an example of a material whose usage is already well established in some important industries, such as aeronautics or aerospace sectors [1,3], as well as in the automotive industry [4]. However, there are multiple different types of damage that can occur during the production and service of the parts produced, such as delamination and matrix cracking, which can cause the failure of the structure in which they are inserted [16,17,18]. Multiple research works have been performed using CFRP as the adherend material in adhesive bonded joints. These works seek to investigate the delamination process, which is caused by the debonding of the fibres and the polymeric matrix due to peel stresses, in order to develop different methods that may help to mitigate this type of failure mode [16,19,20]. Among the techniques developed are z-pinning [21,22], weaving/stitching/tufting [23], or the use of interlayers [19]. Moreover, it is also possible to reduce the occurrence of delamination by reinforcing the adherend with a tough layer of adhesive or a metal [6,19,24].

Fibre metal laminates (FML) are also a promising solution for joint hybridisation. These materials can be used to reduce the delamination process in adhesive joints and they possess good strength to weight properties conferred by the composite part, as well as the damage resistance characteristics and the failure predictability common to metallic materials [14,15,25]. Furthermore, these materials are known to have high fatigue and impact strength, resistance to corrosion, and flame propagation [26]. FMLs are multi-layered structures which contain materials that improve their strength such as aluminium and fibres (aramid, carbon, and glass) [27]. In case there is a crack in one of the layers, the neighbouring layer reduces the stress concentration at the crack tip, which allows to prevent the crack propagation through the whole structure [28]. In addition, the bridging mechanism that is generated around the crack sites is responsible for preventing the proliferation of localised damage within the FML, restricting the rapid propagation of cracks and thus preventing catastrophic failure of the structure [28,29]. Nowadays, the most widely used FMLs, especially in the aeronautical industry, differ mainly in the type of reinforcement fibres of the composite materials and in the type of metal used. In the former, ARRALL (aramid-fibre-reinforced aluminium laminate), CARRALL (carbon-fibre-reinforced aluminium laminate) [26] and GLARE (glass-reinforced aluminium laminate) [30] can be found, while in the latter the main metals used are titanium and aluminium [26]. In practice, the application of some of these materials has been limited by decreasing strength, durability, and reliability, mostly due to corrosion associated with different materials in joining technologies [31]. Morgado et al. [24] studied the use of FML to avoid delamination in a single lap joint (SLJ), combining CFRP and aluminium and varying the metal thickness. The authors showed that the use of metallic laminates in joints allows for the elimination of delamination mechanisms and increases the joint strength, while also attaining an increase in the absorbed energy. It was also concluded that opting for the use of 0.8 mm aluminium sheets increases the weight of the joint by about 15.6%, although it decreases the peel stress at the edge of the overlap. Later, Ramezani et al. [6] showed that the use of 0.4 mm thick aluminium, representing 25% of the total thickness of the laminate, presents a slightly higher failure load than that obtained when using 0.8 mm thick aluminium sheets, representing 50% of the total thickness of the substrate. Although both solutions allow obtaining cohesive failure in the adhesive and a clear improvement in the failure load, when compared with the reference joint using only CFRP, the 0.8 mm option will be the least interesting due to the additional weight it represents in the total joint.

The main ways of bonding composite components are through co-curing, co-bonding, and secondary bonding. In the first, one of the adherends is cured together with the adhesive, while in the second case all parts are cured simultaneously. Generally, these methods are adopted by using pre-preg fabrics and film adhesives, which must be cured at high pressures and temperatures. Finally, in secondary bonding, the adhesive layer is cured between two previously cured adherends [14,32]. The multimaterial bonding method is the same as the secondary bonding method, but instead of using only cured composite substrates, the multimaterial bonding method also uses metal substrates (such as steel, aluminium, titanium, etc.) [32]. Although the thermal strain of the adhesive layer, arising from the manufacturing process [33,34], is often neglected when designing joints, its coefficient of thermal expansion (CTE) is sometimes several orders of magnitude higher than that of adherends, and thus should be considered [35]. Additionally, there is also interest in knowing the residual stress state resulting from the manufacturing process, in composite materials, since they have an influence in the mechanical properties. This is particularly significant for FML [36,37], as when a sheet of metal is cured together with FRP and then cooled to room temperature, the different CTE of each material causes residual thermal stresses to appear [38,39,40]. Furthermore, the difference between curing temperature and operational temperature of the cured laminate also has an influence on the development of these residual thermal stresses. They are primarily influenced by the chemical shrinkage of the plastic and the thermal expansions of the various components, which are both influenced by a diverse variety of extrinsic and intrinsic parameters. Material properties, lay-up arrangements, and the overall volume ratio of metal to composite are intrinsic parameters, while process- and tool-related parameters are extrinsic parameters. As a result, the residual stress state that is created is a complex combination of many different parameters and necessarily requires additional research. These stresses may significantly reduce the hybrid laminate’s mechanical properties, depending on the constituent’s stiffness and fraction, particularly when the difference in thermal expansion is large [41].

Wiedemann et al. [36] used strain gauges applied to the metal layer of FMLs to measure absolute strains during the cure process. The variation in the CTE allowed for the measurement of the cure state of the adhesive layer, independently of the location of the sensors, proving that the method can be simple and accurate. The authors studied different curing cycles and were able to reduce the residual thermal stresses in a CFRP-steel laminate up to 50%. Prussak et al. [41] resorted to fibre Bragg grating (FBG) to measure in-plane strains during the manufacturing of CFRP-steel laminates. During this research, the relationship between the curing reaction and the measured strains could be evidently described, as well as the modification in residual stresses when the process is changed.

It is generally time-consuming and expensive to experimentally measure the residual thermal stresses generated during the curing process. Therefore, it is frequently chosen to predict the values of these stresses using computational tools [42]. Wang et al. [43] compared the results obtained from a finite element model (FEM) characterization of the residual stresses with the experimental layer removal method and, afterwards, the same stresses were inputted in a FEM to predict the mechanical behaviour of an FML and analyse the effects on the tensile and interlaminar shear stresses. The study revealed that the aluminium layers of GLARE had residual stresses in the tensile and compressive directions of about 40 MPa and 70 MPa, respectively. Additionally, the residual stresses had little impact on the tensile test failure mode, although they did slightly lower the tensile strength and initial damage loads. The residual stresses in the adhesive layers of GLARE reduced the peak load and initial damage load during interlaminar shear tests, and this had a minor impact on the interlaminar shear strength. Bellini et al. [44] presented a numerical model to simulate deformations induced by curing in FMLs that was experimentally validated. A notched laminate was used to highlight the deformation displacement, and both numerical and experimental results indicated that the highest deformation occurred near the vertex tip of the laminate. At this location, the opening of the notch was higher than 4 mm.

The present work analyses the influence of residual thermal stresses in SLJ joints using asymmetric CFRP-aluminium (CFRP-Al) adherends. The obtained results are compared with those of symmetric CFRP-only (CFRP) and Aluminium-CFRP-Aluminium (Al-CFRP-Al) joints. The digital image correlation (DIC) method is applied, in order to evaluate the strain evolution along the experimental tests, as well as to compare the deformation obtained in the substrates, after the curing process. Numerically, an elastic model is used to evaluate the peel and shear stresses after curing and to determine the relevance of the solution under study. Furthermore, the same model is used to carry out a comparison between the stress state of the joints. Additionally, a model to predict the joint strength and failure mode is developed, resorting to cohesive zone modelling (CZM).

## 2. Experimental Details

### 2.1. Materials

The materials used throughout this work were chosen based on their relevance for applications in the aeronautical and aerospace sector. The adhesive, high-performance composite, and the aluminium alloys are all consistent with the materials being currently used in aircraft construction.

#### 2.1.1. Adhesive

In the present work, a structural adhesive was used, with the commercial reference Scotch Weld AF 163-2k (3M, Saint Paul, Minnesota, USA). This material belongs to a family of modified epoxies supplied in film form, characterised by high fracture toughness and peel strength. Additionally, it exhibits high bond strength for temperatures from −55 °C to 120 °C, as well as high resistance to high humidity environments before and after curing [45]. The adhesive was cured according to the manufacturer’s recommendations at 130 °C for 1 h. Table 1 presents the material mechanical properties that were reported by Santos et al. [46].

#### 2.1.2. Adherend

The composite material used in all configurations under study was a unidirectional prepreg CFRP, commercially named Texipreg HS 160 T700 (Seal Spa, Legnano, Italy), with a ply thickness of 0.15 mm. This material has orthotropic elastic properties, presented in Table 2, that were previously determined by Campilho et al. [47]. The cohesive properties that were further used in the numerical analysis are presented in Table 3 and were previously obtained in different studies [8,48,49].

For the FML, the aluminium alloy 2024-T3 Alclad series was used for the 0.4 mm thick sheets, supplied by AMI Metals, Charleroi, Belgium. The mechanical properties of this alloy that were needed for the study are provided in Table 4.

### 2.2. SLJ Specimen Manufacturing

**Figure 1:**
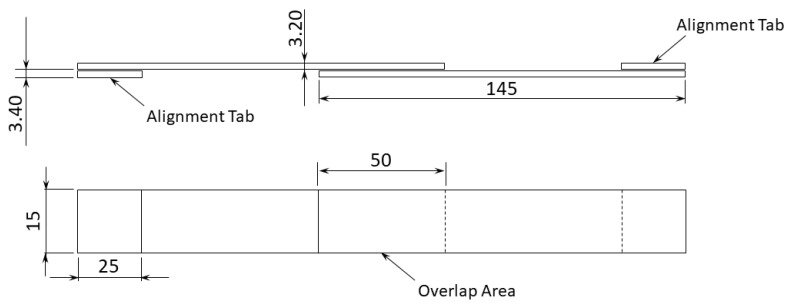
SLJ specimen geometry, in mm.

The specimens’ geometry used to perform the test is depicted in Figure 1.

**Figure 2:**
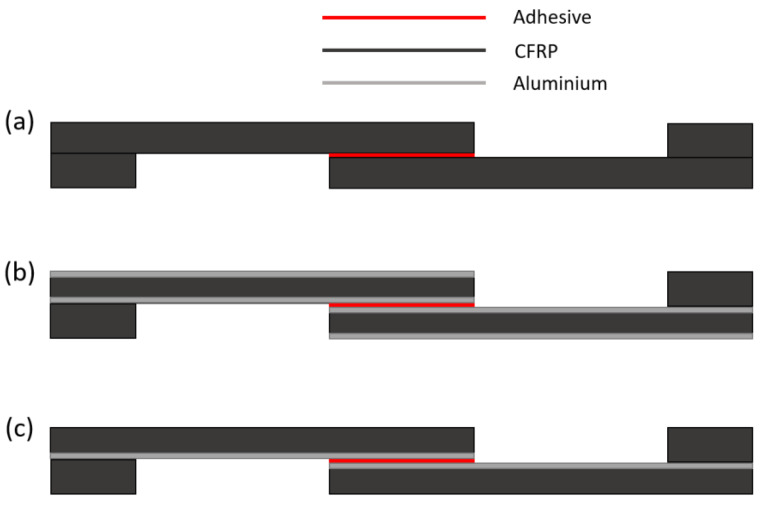
Schematic representation of (**a**) CFRP-only adherend, (**b**) Al-CFRP-Al adherend, and (**c**) CFRP-Al adherend.

The differences between the specimens consisted only in the ratios of material used, as well as in their relative arrangement, with the external geometry and general dimensions remaining unchanged. Thus, as it can be seen in Figure 2, only in the through thickness direction are these changes are visible. Regarding the specimens with FML adherends, the difference between both configurations lies in the number of metal sheets used.

The film adhesive used has a knit support, which allows production with good thickness control. At the time the assembled joints were positioned in the mould, shims were placed on the edges of the mould so that the 0.2 mm final thickness of the adhesive was controlled. Thus, considering that the substrates can be cured under the same pressure and temperature conditions, the complete joint was produced simultaneously, within a single mould. In a first phase, the CFRP was stacked until the desired thickness was reached and, after the assembly of all the parts was completed, the joints were cured in the hot press. In the cases where FML was used, the aluminium metal sheets were added after the CFRP stacking, and the previously described procedure was executed.

For the FML, an additional step had to be performed to ensure good adhesion between the aluminium and the composite. Previous studies [24,50,51] determined that the manual application of a sol–gel-based phosphoric acid to the surfaces in contact with the composite would be sufficient to achieve a superior adhesion. Therefore, the same procedure was performed using the 3M Surface Pre-Treatment AC-130-2 primer on all joints where the laminates were used. Although it is well known that in these laminates the galvanic corrosion phenomenon is prone to occur, the present study is an exploratory work regarding only the geometry and mechanical properties of the joints under study, and thus this issue was not addressed.

Finally, to allow for a subsequent DIC analysis, the specimens for all configurations were coated with white matte paint and speckled with black ink dots.

### 2.3. Testing Setup

The quasi-static condition SLJ tests were performed on a universal machine INSTRON^®^ 3367 (Illinois Tool Works, Hopkinton, Massachusetts, USA), equipped with a 30 kN load cell. All tests were performed at a constant crosshead speed of 1 mm/min, as indicated in ASTM D5868-01(2014) “Standard Test Method for Lap Shear Adhesion for Fiber Reinforced Plastic (FRP) Bonding” [52]. The specimens were fixed using clamps that held their free extremities and four bolts that were manually tightened with a torque wrench. Up to 30 N·m of torque was gradually applied, ensuring an even clamping force. The sample and the clamps were lined up with dowel pins.

To allow for subsequent 2D DIC analysis, it was guaranteed that the acquisition of the load applied to the specimens would be synchronized with the images acquired to register the displacement field. Thus, the data acquisition performed by the testing machine was synchronized with the images acquired by a Nikon D5300 digital camera (Tokyo, Japan), where a Nikon AF-P NIKKOR 18–55 mm f/3.5–5.6 lens was attached.

## 3. Numerical Details

The developed numerical models aimed firstly at understanding the behaviour of the CFRP-Al joints under analysis, in a phase immediately after their curing. Then, a second model was developed, one that could predict the failure load and the failure mode of the joints when tested under quasi-static conditions. All the numerical models were developed using ABAQUS software and were based on 2D planar deformable shell parts.

### 3.1. Elastic Model

**Figure 3:**
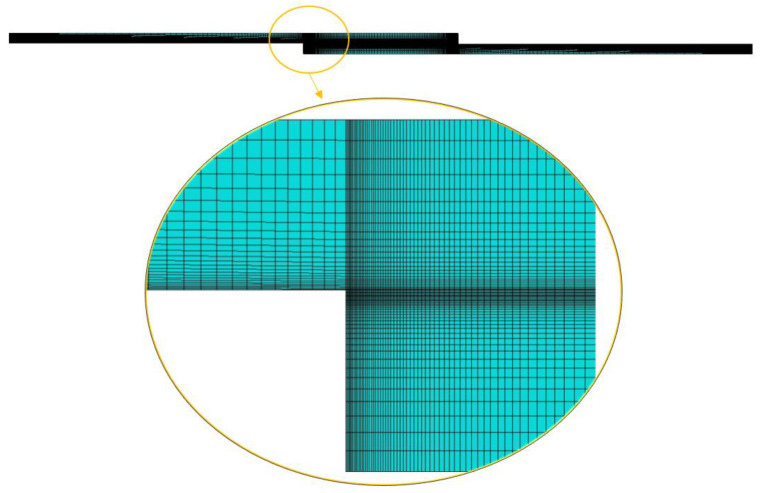
Geometry and mesh of the elastic model.

An elastic model was first developed, using a mesh with four-node plane strain (CPE4R) elements, as shown in Figure 3. No boundary condition was defined and only a predefined temperature field was applied, representative of the difference between the curing temperature and a room temperature of 110 °C. After completion of this simulation with just the thermal step, for the CFRP-Al geometry, where the deformation is clear, it was possible to make a comparison between the predicted deformation value and the one obtained after curing.

**Figure 4:**
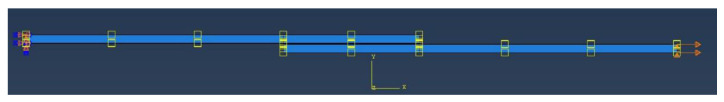
Geometry and boundary conditions of the SLJ model.

Then, for the same elastic model, a step was added to simulate the stresses to which the joints were subjected during the experimental tests. Thus, as can be seen in Figure 4, one end of the SLJ was restricted in all directions while the opposite end was restrained in the vertical direction, and a displacement was applied in the horizontal direction. This analysis made it possible to analyse, along the adhesive layer, the evolution of the peel and shear stresses, as well as to compare the stress state between the different joints.

### 3.2. CZM Model

A CZM model was developed to simulate the adhesive behaviour, predicting its failure load and critical displacement. A triangular tensile-separation law was used to model the adhesive layers, to simulate the damage evolution in these layers. Besides the adhesive, a cohesive layer was also added to the CFRP substrate, allowing to predict delamination phenomena. These elements were positioned between elements of elastic layers of the CFRP, their position depending on the type of modelled geometry.

**Figure 5:**
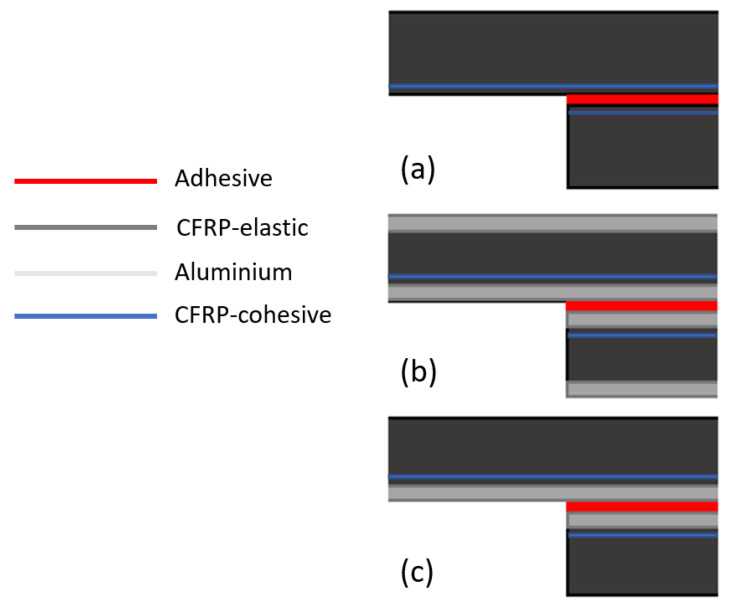
Section assignment for: (**a**) CFRP, (**b**) Al-CFRP-Al, and (**c**) CFRP-Al.

In the case of the CFRP simulation, the cohesive layer, with a thickness of a half ply of the composite, i.e., 0.075 mm, was also positioned at a distance of 0.075 mm from the adhesive layer. For the remaining two geometries, the same layer was positioned above the aluminium layer, also at a half ply distance of the composite. Additionally, the elastoplastic behaviour of the aluminium was also modelled. Figure 5 depicts the different sections used in the model.

The different sections were modelled with elements appropriate to the material being represented, namely: four-node plane stress (CPS4R) elements for the elastic CFRP and four-node plane strain (CPE4R) elements for the aluminium. Cohesive sections were modelled using cohesive elements (COH2D4).

## 4. Results and Discussion

### 4.1. Deflection after Curing

**Figure 6:**
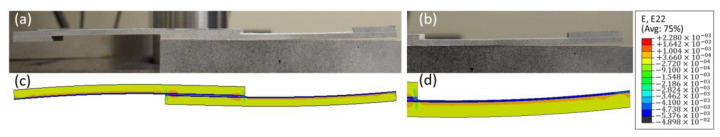
SLJ deformation after curing: (**a**) specimen produced before DIC analysis (and detail (**b**)) and (**c**) specimen model after thermal step (and detail (**d**)).

The deflection exhibited by the CFRP-Al substrates, after the curing process, was experimentally measured and then compared with the numerical results. By using the DIC technique, it was possible to compare the joint performance in both cases. Figure 6 demonstrates an example of an SLJ ready for analysis, as well as the result of the prediction of the numerical model, regarding the expected deflection. The displacement was measured at the edge of the joint, relative to the central point of the overlap, where there should be no deflection.

From what can be observed in Table 5, the results measured by DIC and the prediction of the numerical simulation show a good correlation, which provides evidence for the accuracy of the model. The edge of the joints was displaced about 2 mm in the vertical direction, which is a considerable value, given the size of the joint. Although this deformation may contribute to a decrease in the value of the peel stresses, during the joint test, its application in larger structures and with more complex geometries may be difficult to achieve.

### 4.2. Elastic Modelling Analysis

The displacement of the elastic model was adjusted to impose a maximum principal stress corresponding to that leading to the failure of the CFRP reference joints. The aim was to analyse the ultimate stress state of each joint when compared with the reference geometry. For this purpose, a nodal path was defined in the numerical model and used to obtain the numerical results of the stresses under analysis, along the joint overlap.

**Figure 7:**
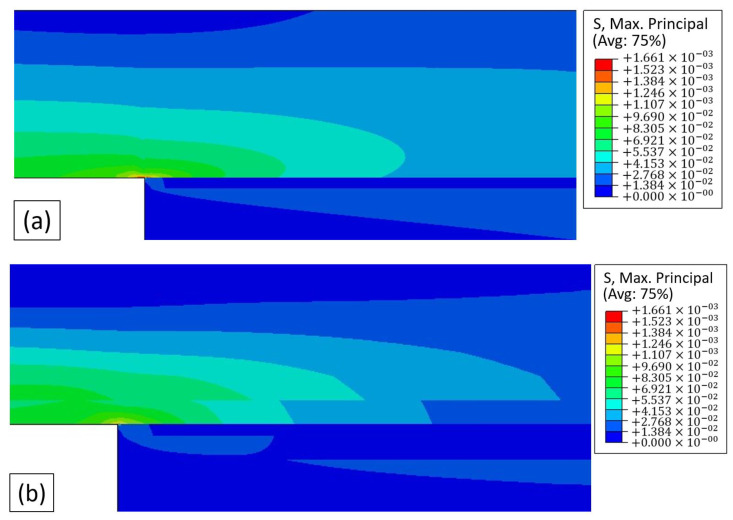

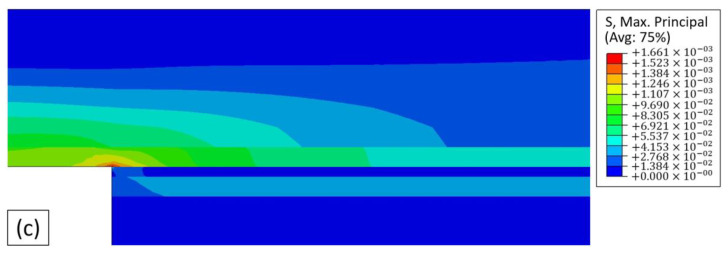
Maximum principal stress distribution for: (**a**) CFRP, (**b**) Al-CFRP-Al, and (**c**) CFRP-Al.

Figure 7 represents the stress distribution near the overlap, considering a maximum principal stress analysis. It is possible to verify that, for the maximum displacement of CFRP specimens, the three geometries present visibly different stress distributions. The introduction of aluminium in the substrates is able to redistribute stresses, minimizing the appearance of localised stress distribution. This more localized concentration of stresses, which in the case of CFRP is near the edge of the bondline, corresponds to the location where joint failure by delamination is expected to occur.

**Figure 8:**
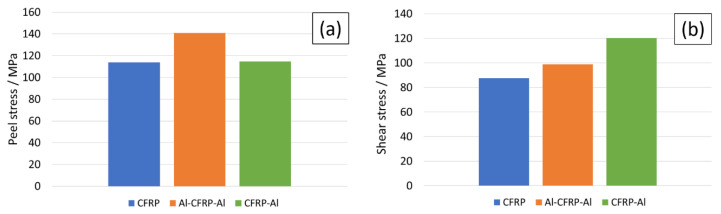
Maximum values for (**a**) peel stress and (**b**) shear stress for all the geometries.

In addition to the study of the maximum principal stresses, the maximum peel and shear stresses in the adhesive layer were also analysed using this model. Figure 8a evidences the advantage that the existence of the residual thermal stresses can have. For the displacement predicted for the CFRP failure, where failure occurs via delamination, this joint presents the same value for peel stress, although it does not present joint failure. Since the edges of the overlap are under a compressive state after curing, loading this joint up to this displacement develops less peel stress in this zone, which increases the load-bearing capability of the joint. Although the Al-CFRP-Al joint presents higher peel stress values, the aluminium foil is responsible for transferring part of the load, which redistributes the load along a larger area and thus avoids the delamination process. As for the shear stresses, Figure 8b shows that they are lower for the reference geometry, increasing progressively for Al-CFRP-Al and CFRP-AL configurations. However, these values have a less preponderant influence on the joint since it is the loading mode under which the joint is more resistant. It is therefore the performance under peel behaviour that can determine the success of a given SLJ configuration. It is to be noted that, at this stage of the analysis, the CFRP configuration will fail, while for the other two configurations the stress values are still far from the strength of aluminium, which is the main factor responsible for the load transfer in the joint.

### 4.3. SLJ Results

**Figure 9:**
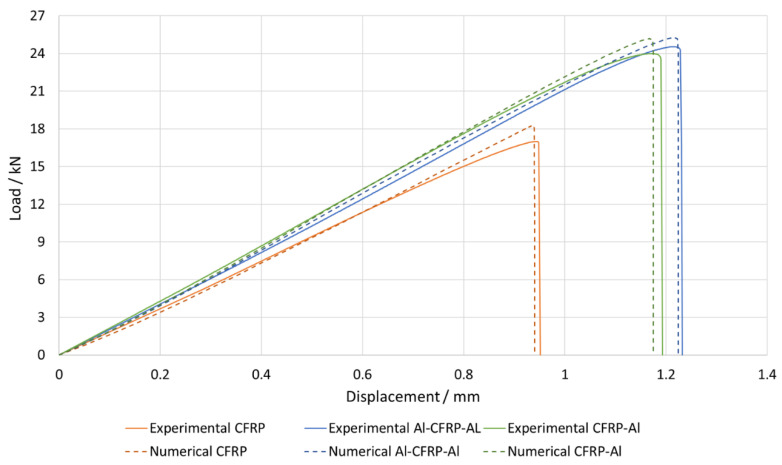
Experimental versus numerical representative load–displacement curves of the different tested geometries.

Joints of all configurations produced were subjected to tests under quasi-static conditions, allowing their failure load and critical displacement to be analysed. Figure 9 illustrates that the use of FML allows for an increase in both failure load and the critical displacement. The presented experimental curves were chosen as the most representative for each configuration. Regarding the numerical analysis, a good level of correlation was generally found, with the difference between their failure loads never exceeding 10%. Furthermore, the experimentally observed failure mode was also reproduced correctly.

Knowing the strength of the joints, their performance in relation to their specific strength was analysed, given that the weight of the joint may play an important role in the decision to use them in structural parts. It was interesting to verify that the reference joint, CFRP, presented the lowest average specific strength value of 74.27 kN/kg. The Al-CFRP-Al solution presented the intermediate value of 86.4 kN/kg, while the CFRP-Al solution presented the best performance with the highest value of 92.43 kN/kg.

**Figure 10:**
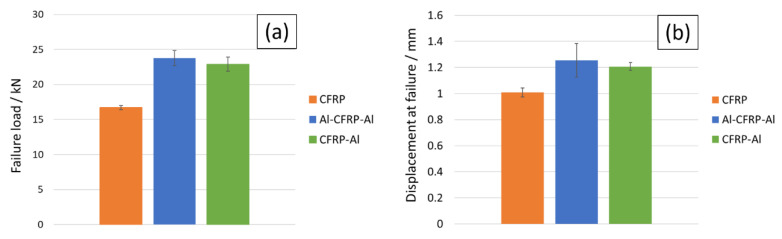
Different geometries’ failure values for experimental load (**a**) and displacement (**b**).

Although the CFRP-Al curve in Figure 9 shows a lower failure load than the Al-CFRP-Al result, by analysing Figure 10, it can be concluded that this difference is within the standard deviation range, and thus it can be stated that both solutions present a similar performance. Although the Al-CFRP-Al joint allows for a less localised stress distribution, the thermal residual stresses seem to compensate for the difference in stress distribution, and thus match the performance of the symmetrical solution.

Joints reinforced with aluminium presented a strength gain above 35% for both cases when compared with the reference CFRP joint, which presented a base strength of 16.71±3.07 kN. The Al-CFRP-Al joint reached a strength value of 23.76±1.08 kN, representing a 42.2% gain, while the CFRP-Al joint demonstrated a failure load value of 22.90±1.01 kN, representing a 37.1% gain, when compared with the reference value. Regarding the numerical analysis, the model for the CFRP configuration outputted a failure load 9.4% above the average value, while the Al-CFRP-Al and CFRP-Al configurations returned deviations of 6.4% and 9.9%, respectively.

#### Failure Mechanism Analysis

**Figure 11:**
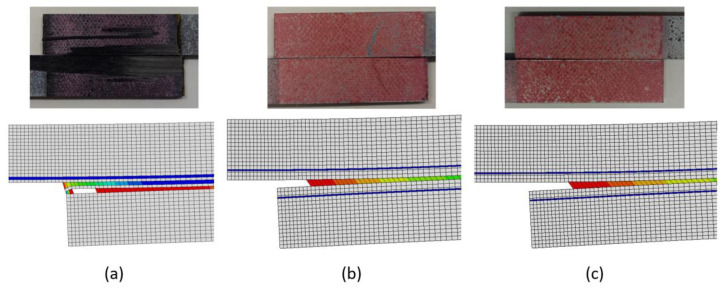
Experimental and numerical failure mode of the different geometries: (**a**) CFRP, (**b**) Al-CFRP-Al, and (**c**) CFRP-Al.

Figure 11 depicts the failure modes obtained for the different joint types. It can be observed that FML joints, regardless of the configuration, allow delamination failure to be avoided. The aluminium sheets lead to a smoother stress distribution along the adhesive layer and a lower stress concentration at the edges of the overlap.

Additionally, as it is evidenced in Figure 11, the failure mode was predicted correctly by the numerical models. It can be observed that, in the case of CFRP, joints fail by delamination, as reported experimentally. However, the model seems to indicate that the adhesive would also be already damaged at this stage. Thus, in the case of this geometry, there seems to be competition between both modes, with some cohesive failure already present at the end of the test. This may explain why in some of the experimental tests there is evidence for some cohesive failure, although delamination is clearly the dominant mode. For the FML joints, the presence of cohesive failure is evident and there is no delamination damage.

**Figure 12:**
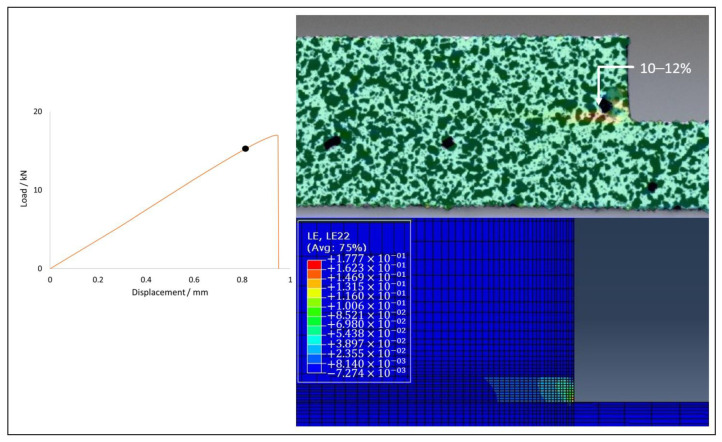
Strain field in CFRP joint: DIC (top) vs. numerical (bottom).

**Figure 13:**
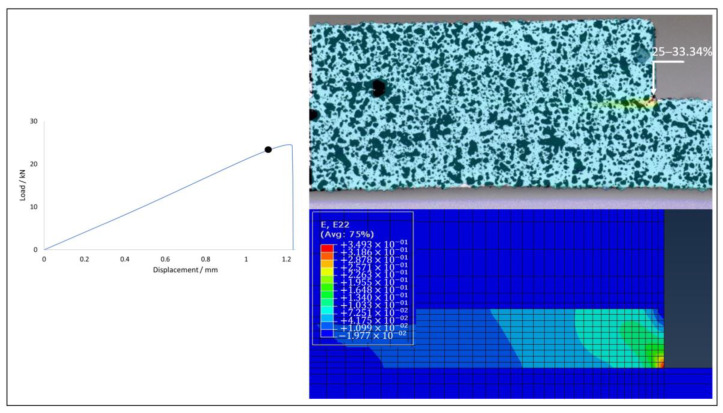
Strain field in Al-CFRP-Al joint: DIC (top) vs. numerical (bottom).

**Figure 14:**
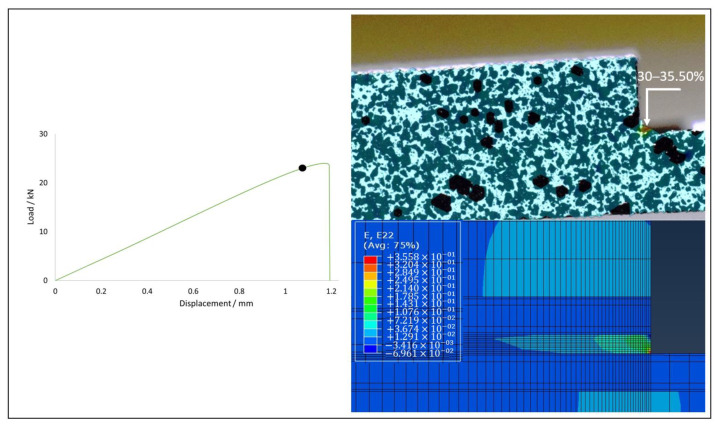
Strain field in CFRP-Al joint: DIC (top) vs. numerical (bottom).

The prediction of the strain level present in the moments preceding the crack propagation and subsequent failure of the specimen were found to be accurate when compared with the strain fields determined using the 2D DIC analysis. Larger strain values are very localized and, therefore, restricted to a very small area. Although measurements taken with the digital extensometer present a scale with lower resolution, it was still possible to visually identify the moment prior to crack propagation. The comparison between experimental and numerical data proved useful to understand how deformations develop in the critical zone. Figure 12, Figure 13 and Figure 14 show a good level of agreement for both methods when the deformation in the vertical direction is analysed. By observing the results of the three geometries tested, it is clear that the CFRP specimen undergoes less deformation before failure, which is in accordance with the delamination failure mode that it exhibits. However, in this case the adhesive layer, which is the one presenting the most deformation, has very localised displacements and, therefore, the digital extensometer has significant difficulties in registering this phenomenon. Thus, the experimental and numerical values show some difference. All specimen configurations using FML substrates presented strains of the same order of magnitude, a phenomenon that is corroborated by the values obtained in the failure load. The use of aluminium in the layup allows this material to deform during loading, reducing the concentration of stresses at the edge of the overlap and, therefore, guaranteeing cohesive failure in the adhesive and not delamination, which is the most desirable failure mode for these connections.

## 5. Conclusions

The influence of the residual thermal stresses induced by the curing process in the mechanical performance of CFRP-Al SLJs, was assessed in the present work. To analyse the failure load and the failure mode of the configurations under study, tests were performed in quasi-static conditions and these results were compared with those stemming from a set of numerical models. All results were compared with a reference composite joint, as well as with a second FML joint, manufactured with symmetric Al-CFRP-Al substrates. Since the latter uses symmetric substrates, the curing process does not produce any kind of relevant residual stress for the geometry under study.

- The **CFRP-Al** specimens showed a **large deviation** from its initial geometry **after curing**, which may be unacceptable for larger and more complex geometries. However, the controlled use of these deformations, adapted to each application, may prove to be advantageous.
- The values obtained for **joint strength** showed that the smoother stress distribution achieved by the introduction of **aluminium layers** has a **positive influence** on the performance of composite joints. These layers were found to be able to avoid failure by delamination and to increase joint strength by **more than 35%**.
- Although both FML configurations exhibited similar performance, both in joint strength and failure mode, the **CFRP-Al** solution presented the **highest specific strength**. This is of importance since these materials are to be used in the aeronautical and aerospace industries, where high specific strength is imperative.
- All **numerical models** presented good correlation with the experimental results. For all joints, the numerically determined **failure load** values were generally **close to the experimentally** determined **values**.
- The **failure modes** obtained experimentally were also **correctly reproduced** by the **models**.

The present work allowed the development of a configuration that may be promising in aeronautical and aerospace applications. The fact that the joint under study presents a specific strength higher than the reference joints makes its use in environments where the weight of the structures is important a possibility. However, there are issues that should be addressed in future work, such as the limitations that deformations may present and the fact that this combination of materials is prone to galvanic corrosion.

## Figures and Tables

**Table 1:** 3M AF 163-2k mechanical properties [24,46].

Property	Value
Young’s Modulus [GPa]	1.52±0.12
Tensile Strength [MPa]	46.93±0.63
Shear Modulus [MPa]	567.86
Shear Strength [MPa]	46.86±2.57
*G_IC_* [N/mm]	4.05±0.07
*G_IIC_* [N/mm]	9.77±0.21

**Table 2:** CFRP elastic orthotropic properties [47].

*E_x_* (MPa)	*E_y_* (MPa)	*E_z_* (MPa)	*ν* *_xy_*	*ν* *_yz_*	*ν* *_xz_*	*G_xy_* (MPa)	*G_yz_* (MPa)	*G_xz_* (MPa)
109,000	8819	8819	0.342	0.342	0.38	4315	4315	3200

**Table 3:** CFRP cohesive properties [8,48,49].

Property	Value
$t_{n}^{0}$ [MPa]	40
$t_{s}^{0}$ [MPa]	35
*G_IC_* [N/mm]	0.59
*G_IIC_* [N/mm]	1.2

**Table 4:** Mechanical properties of the 2024-T3 aluminium alloy [24].

Young’s Modulus (GPa)	Poisson’s Ratio
66	0.3

**Table 5:** Comparison between curing displacements of the specimens: experimental vs. numerical.

Average Displacement Measured with DIC	Displacement Measured by the Numerical Analysis
2.05 ±0.08 m	1.84 mm
